# Voltage imaging in zebrafish using high-speed light-sheet microscopy

**DOI:** 10.1117/1.NPh.13.S2.S23204

**Published:** 2026-03-25

**Authors:** Urs L. Böhm, Zeguan Wang, Takashi Kawashima

**Affiliations:** a Université Paris Cité, Institute of Psychiatry and Neuroscience of Paris (IPNP), INSERM U1266, Paris, France; b McGovern Institute for Brain Research, Massachusetts Institute of Technology, Cambridge, Massachusetts, United States; c Weizmann Institute of Science, Department of Brain Sciences, Rehovot, Israel

**Keywords:** voltage imaging, light-sheet microscopy, zebrafish

## Abstract

Voltage imaging in small model animals, such as larval zebrafish, has opened new avenues for understanding how millisecond-scale population neural dynamics drive behaviors. In these animals, multielectrode insertion is technically infeasible, and voltage imaging is the only viable approach for recording spiking activity from many neurons simultaneously. At the same time, the combination of brain transparency and high-speed light-sheet microscopy provides a unique opportunity to apply this technology not only to the brain surface but across the entire brain and spinal cord. Here, we review recent technological advances and neural circuit discoveries made using this technology.

## 1 Introduction

Zebrafish have been an essential model organism for understanding the development and function of neural circuits that drive complex behaviors.^1^^–^^4^ Larval zebrafish hatch from eggs at 3 or 4 days after fertilization (d.p.f.) and immediately exhibit complex behaviors, such as escape,^5^ prey capture,^6^^–^^8^ optomotor response,^9^ motor learning,^10^^,^^11^ sleep-wake cycle,^12^ and associative learning.^13^ Their larval brains are small (0.5 mm in width, 1 mm in length, and 0.3 mm in depth) and translucent until the juvenile stage, a few weeks after fertilization. This developmental window offers optical access to the brain and facilitates circuit-level imaging and perturbation.

Neural activity imaging in living vertebrates was first demonstrated in the zebrafish spinal cord using a synthetic calcium dye,^14^ following its earlier demonstrations in invertebrates.^15^ Neural activity imaging in zebrafish was expanded in scale using improved genetically encoded calcium indicators^16^^,^^17^ and mutants with less pigmentation.^18^^,^^19^ The introduction of scanned light-sheet microscopy^20^ established whole-brain neural activity imaging methods.^10^^,^^11^^,^^21^ This technology allowed the simultaneous recording of neural activity from almost all neurons in the brain at single-cell resolution and significantly expanded our understanding of how brain-wide neural activity drives various behaviors.^22^^–^^25^

Insights from these large-scale neural activity imaging methods, however, were fundamentally limited because neuronal calcium dynamics are significantly slower than their spiking dynamics. Single-cell extracellular and intracellular recordings^11^^,^^26^^–^^32^ revealed millisecond-timescale neural computations in the larval zebrafish brain. Nonetheless, these recording techniques were highly invasive due to the relative sizes of the electrodes in the small zebrafish brain. For example, the electrode diameter of 20  μm scales to 3 mm for the human brain. The use of larger multielectrode arrays^33^ would be even more invasive. In addition, the small size of zebrafish neurons (5  μm in diameter) limited the success rate and recording durations, as subtle brain drifts from surgical deformation and tail movements can cause the electrodes to detach. Thus, insights into fast neural dynamics in the larval zebrafish brain remain limited.

**Fig. 1:**
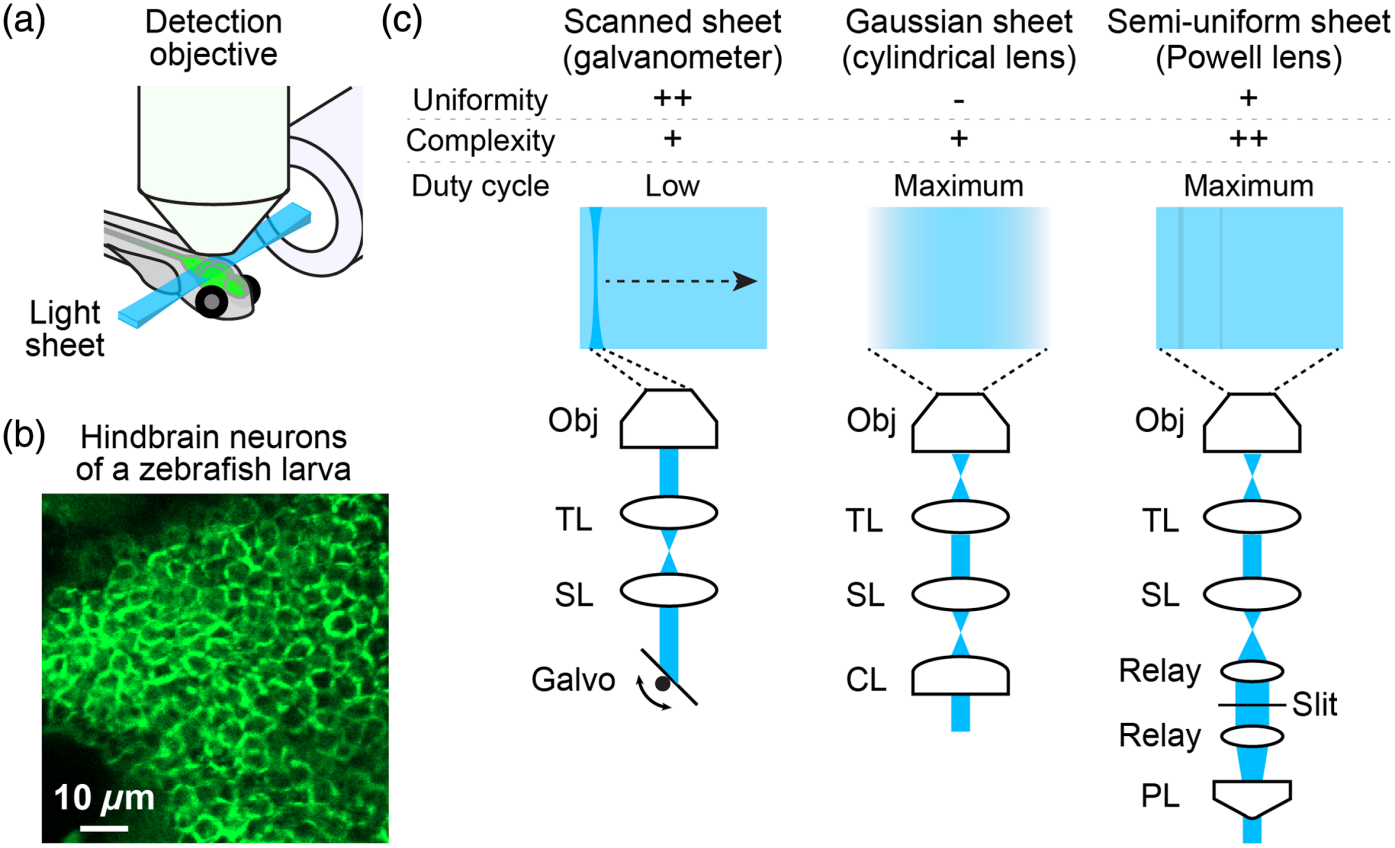
Light-sheet microscopy for voltage imaging in zebrafish. (a) Light-sheet microscopy of the zebrafish brain. The horizontal light sheet is generated from the excitation objective at the axial plane of the detection objective, through which a high-speed camera acquires fluorescence images. (b) Densely packed neurons in the hindbrain of a 6-day-old zebrafish larva. Membrane-bound Voltron indicator was expressed pan-neuronally and conjugated with a fluorescent dye. (c) Different modes of light sheet generation. Their pros and cons for sheet uniformity, complexity of optical paths, and spatial duty cycle are listed. The light sheet generated by a Powell lens is usually not perfectly uniform because of subtle irregularities of the tip of the Powell lens. Objective lens (Obj); tube lens (TL); scan lens (SL); galvanometer (Galvo); cylindrical lens (CL); relay lens (Relay); Powell lens (PL). Placing a slit between the relay lens enhances the quality of the light sheet after the objective.

The recent development of genetically encoded voltage indicators has opened new opportunities for recording neural activity at millisecond timescales in a noninvasive manner. Light-sheet microscopy, in principle, allows the speed of up to a thousand frames per second in any place in the zebrafish brain [Fig. 1(a)]. Here, we review the recent developments and neural circuit insights obtained by voltage imaging in zebrafish.

## 2 Use of Genetically Encoded Voltage Indicators

Voltage imaging using genetically encoded voltage indicators (GEVI) has the potential to overcome the above difficulties for electrophysiology in the zebrafish because nothing needs to be inserted into the brain, and small drifts can be corrected through post-hoc image registration. Moreover, it can record from a large number of neurons simultaneously.

Nonetheless, voltage imaging presents unique challenges when applied to the zebrafish brain. First, the small neuronal size (∼5  μm) significantly limits the photon budget per neuron.^34^^,^^35^ The number of photons N collected per neuron in a single frame determines the shot noise level (1/√N), which directly affects the signal-to-noise ratio (SNR) for spike detection. Because somatic surface area is proportional to the square of neuronal size, achieving the same SNR in zebrafish requires roughly fourfold higher excitation light than for a similarly bright, large pyramidal neuron in the mammalian cortex (∼10  μm) [Realistically, we estimate that the brightness differences are somewhere between two and four times. If we assume an ideal optical section of a neuronal membrane as a circle, the difference is two times. However, the typical waist size of the excitation beam of a light-sheet microscope for zebrafish neural activity imaging is around 5  μm (full width half maximum), which is almost equal to the diameter of larval zebrafish neurons. Considering the additional optical sectioning provided by the detection objective, we estimate that the true brightness differences lie between the ideal optical sectioning (two times) and epifluorescence (four times)]. Strong excitation light bleaches fluorophores quickly, limiting the duration of voltage imaging. A bright and photostable GEVI is therefore necessary to overcome this problem.

Second, neuronal somata in the larval zebrafish brain are tightly packed and separated by sub-diffraction distances^36^^,^^37^ [Fig. 1(b)]. As voltage signals localize to the plasma membrane, it is necessary to sparsely express voltage indicators to segment signals between neurons. Pioneering studies of voltage imaging that pan-neuronally expressed GEVIs, such as ASAP1^38^^,^^39^ and Bongwoori,^39^ revealed membrane potential fluctuations at a regional level but did not resolve individual spiking activities. New studies achieved sparse expression of another GEVI, Archon,^40^ using either transient transgenics that mosaically express egg-injected plasmids^40^ or transgenic zebrafish that only express it in specific cell types.^41^ These sparse expression strategies enabled the first recording of the spiking activity from multiple neurons and axons in the spinal cord.^40^^,^^41^ Thus, sparse expression is crucial for enhancing the SNR of voltage imaging in zebrafish.

The invention of chemigenetic voltage indicators, Voltron, significantly extended the recording duration of voltage imaging in behaving larval zebrafish.^42^ This rhodopsin-based indicator modulates the brightness of a covalently-bound synthetic dye^43^ using Förster resonance energy transfer (FRET). The indicators are expressed in genetically targeted neural populations and, after the bath application of the dye to zebrafish, are tagged with the dye via HaloTag^44^ mechanism. The superb brightness of the synthetic dye compared with past GEVIs allowed orders-of-magnitude lower brightness of the excitation light, which was crucial for the fish’s task performance during the imaging. Its photostability enabled the stable recording of voltage signals over 10 min while still collecting sufficient photons for spike detection. The use of HaloTag-dye coupling also enabled imaging of distinct neural populations in different wavelengths based on timing mechanisms, such as developmental birth dates.^45^

## 3 Tweaking the Light Sheet for Voltage Imaging

The relatively simple mechanism of light-sheet microscopy is ideally suited for voltage imaging, where the “sheet” of the excitation beam illuminates a specific axial plane, and an objective lens and a camera acquire the fluorescence image of the same axial plane. The imaging speed of this setup is limited only by the camera and is significantly faster than that of point-scanning confocal or two-photon microscopes while still providing high resolution.

Nonetheless, in addition to the above consideration of shot noise, minimizing device noise is crucial. The primary source of device noise is the fluctuation of the brightness and position of the excitation beam. Such efforts include operating continuous-wave (CW) lasers in nonpulsed mode within a power-stable range without frequent on/off switching and using a stable analog supply to power galvanometers for adjusting light-sheet positions (Table 1).

**Table 1:** Common pitfalls for voltage imaging that result in poor signal-to-noise ratio.

Device	Pitfall	Improvement
Any device and command cables	Placed near power strips	Move the power strip or put the power strip in a Faraday cage
	Non-coaxial cables	Use coaxial cables or wrap them with grounded aluminum foils
	Cable coil amplifying electromagnetic noise	Minimum-distance wiring
Laser	Frequent turn on/off	Use continuous illumination
	Operating with too low/high power (<10% or >90% maximum)	Operate in the middle range. Use ND filters for adjusting intensity.
	Air flow from the air-conditioning or device fan on the light path	Shield from excessive flow of cool or hot air
Galvanometer	Using a switching power supply	Use a linear power supply with a large capacitor
	Humming noise	Adjust the notch filter of the galvo controller
	The input voltage range is small relative to the DAQ outputs	Avoid noisy DAQ outputs in a small dynamic range and instead use a scaling amplifier or voltage divider to descale
	Small angular movement adds noise to the light-sheet z-position	Change the scan lens – tube lens combination on the excitation light path
Camera	Not enough photon counts per cell	Calculate photon counts.
		10,000 photons = 1% shot noise.
	The camera is vibrating	Use solid mounts, lower fan speed (in specific models)
	Mismatch between the camera clock and the external frame trigger leads to non-uniform exposure	Use the camera clock to control timing, or use free-running mode
	Readout noise is high	Increase camera analog gain, unless pixels are saturated; switch camera models
	Background is high	The background should be as low as the digitizer offset. Implement tight light isolation around the microscope.
	Excitation light leaks into fluorescent images	Add a dichroic mirror or a second absorptive filter in the emission path.
Indicator expression	Weak GEVI expression	Switch to a stronger promoter; allow enough time to express GEVIs
	Bright GEVI expression but no/little activity	Reduce expression strength; confirm GEVI trafficking and cell health

The spatial duty cycle of the microscope, which determines how long each neuron is illuminated by the excitation beam during a single camera frame, is another factor that significantly affects the detection of spiking activity^35^ [Fig. 1(c)]. In the most common form of the scanning light sheet microscope, the excitation beam sweeps parallel to the detection objective’s axial plane, forming a light sheet. This sweep typically occurs once per frame during the camera exposure. In this case, a cell is illuminated for only a fraction of the camera exposure time. Such a low duty cycle results in poor SNR for spike detection.^35^

Therefore, studies that recorded spiking activities in neurons increased the spatial duty cycle by modifying the shape of the excitation beam while preserving axial resolution. Earlier studies widened the excitation beam in the sweeping direction several times,^42^^,^^46^ whereas later studies created a stationary light sheet using either a cylindrical lens^41^ or a Powell lens^47^ [Fig. 1(c)] as described in the following section. The latter configurations maximize the duty cycle to 1 and offer a significant advantage of light-sheet microscopy for voltage imaging over other types of fluorescence microscopy with lower duty cycles, such as confocal and two-photon microscopy.

## 4 Single-plane Voltage Imaging in Zebrafish

### 4.1 Motor Command and Efference Copy in the Midbrain

**Fig. 2:**
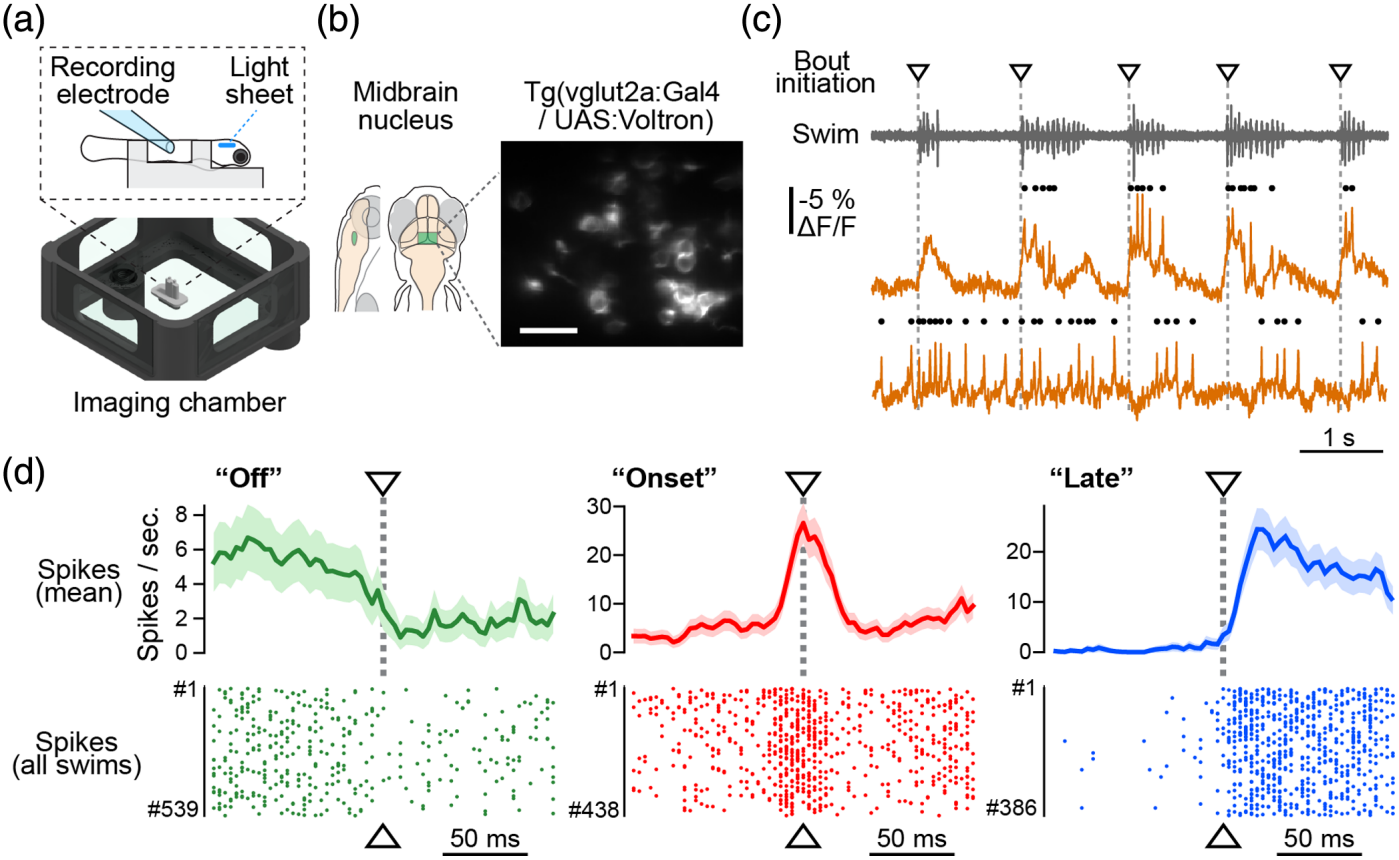
Voltage imaging in behaving zebrafish using a chemigenetic Voltron indicator. (a) Schematics of the imaging chamber for light-sheet microscopy and how we conducted voltage imaging in the brain and motor signal recording in the tail simultaneously. (b) Left, anatomical location of the imaged midbrain nucleus. Right, a representative image of 11 Voltron-expressing neurons with detectable spiking activity. Scale bar, 20  μm. (c) Swimming signals in the virtual reality (top) and Voltron fluorescence traces from two representative neurons (bottom). Dots on the top of each trace represent detected spikes. Downward triangles and dotted gray lines indicate the initiation of each swim bout. (d) Mean spiking frequency (top) and raster plots (bottom) at the initiation of swim bouts from three representative neurons: “Off” (green), “Onset” (red), and “Late” (blue) neurons. Shadows in the top and middle panels represent the standard error of the mean across swim events. Adapted from Abdelfattah, Kawashima, et al.^42^

Voltage imaging using Voltron^42^ was first demonstrated in zebrafish swimming in a virtual reality system [Fig. 2(a)]. Larval fish were placed in a custom imaging chamber that was designed for a canonical light-sheet microscope setup with a low-NA excitation objective and high-NA detection objective.^48^ Fish were immobilized by blocking neuromuscular transmission pharmacologically. Instead, a pair of electrodes attached to the tail recorded ventral root signals, which reflect the collective activity of spinal motoneurons during swimming. Visual stimuli were projected beneath the fish to elicit swimming by optomotor response (OMR). Voltage imaging was performed simultaneously in the brain using a light-sheet microscope with a modified beam shape as described above to increase the spatial duty cycle.

The Voltron indicator was sparsely expressed in *vglut2*+ neurons in the midbrain tegmental areas [Fig. 2(b)]. The sparsity was obtained by selecting a transgenic zebrafish Tg(UAS:Voltron) that drives the transgene sparsely. During Tol2-mediated transgenesis,^49^ the above transgene was randomly integrated into the genome, and the degree of sparsity varied across injected strains due to genomic positional effects and the partial methylation of the UAS promoter.^50^ By selecting between injected strains, it was possible to identify a strain with a sparse transgene expression ideal for voltage imaging.

Using these methodologies, membrane potential and spiking activity were successfully recorded from a dozen neurons in behaving zebrafish [Fig. 2(c)]. Further analyses revealed three types of neural activity around the onset of swimming: “off” types that are suppressed by swimming, “onset” types that are transiently activated around the onset of swimming for 20 to 30 ms and then silenced, and “late” types that are synchronized to swimming but delayed from the spinal cord signal [Fig. 2(d)]. The latter two types potentially correspond to motor command signals and efference copy signals, respectively. The interactions of these signals in the same excitatory cell types within the same brain nuclei demonstrate the complexity of motor command generation in the zebrafish brain.

### 4.2 Modulation of Motor Output in the Spinal Cord

**Fig. 3:**
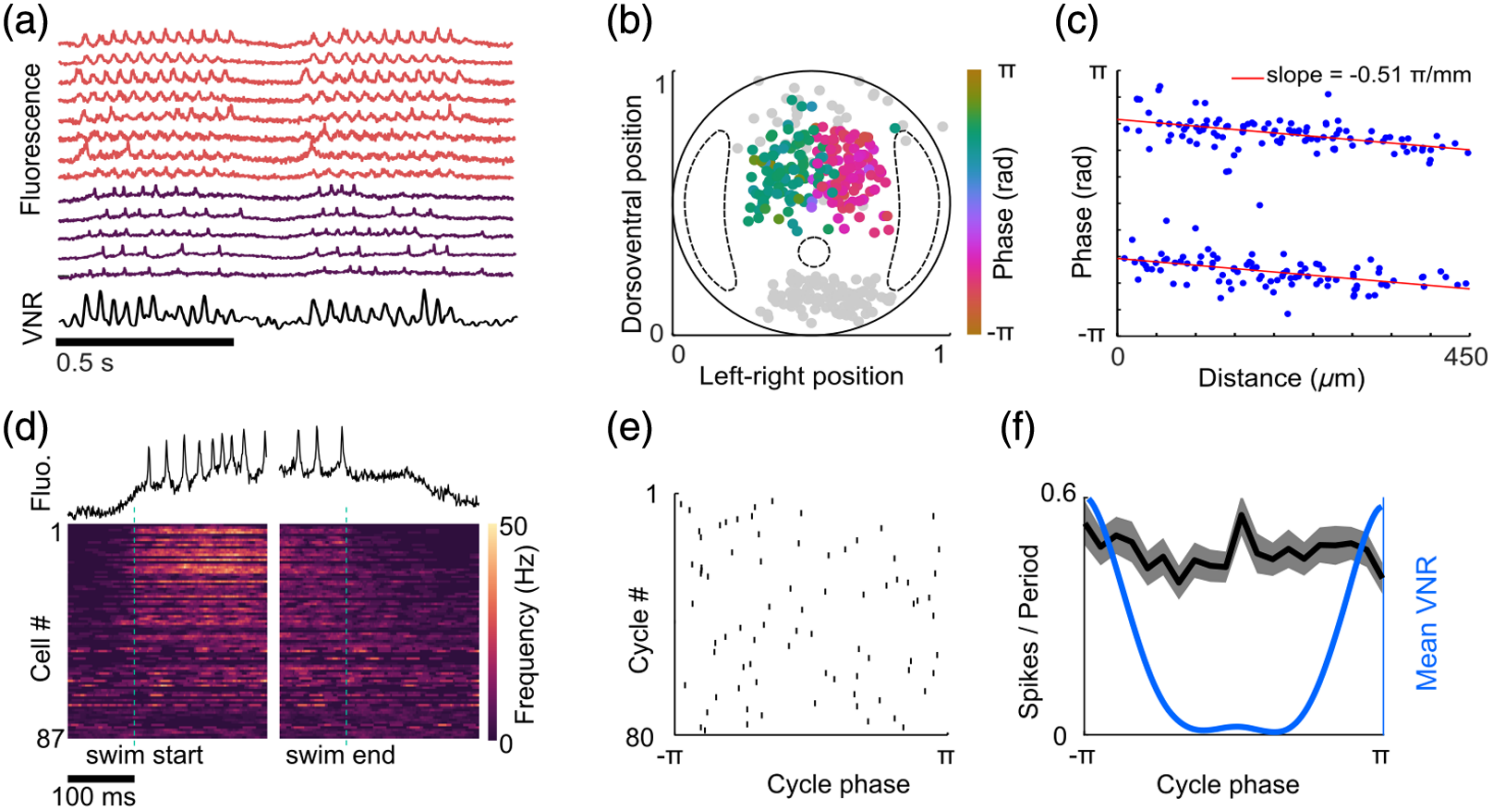
Oscillating and non-oscillating spinal cord neurons. (a) Example traces of recorded oscillating (red) and nonoscillating (purple) neurons together with fictive motor output ventral nerve root (VNR). (b) Transverse view showing cell-body positions of oscillating neurons, color-coded by phase relative to VNR. Nonoscillating cells in gray; dotted lines indicate the position of the lateral neuropil region and the central canal. (c) Relationship of average phase and cell body position of oscillating cells along the rostro-caudal axis. The two populations indicate cell bodies on the left and right sides of the spinal cord. The slope indicates the phase delay along the tail. (d) Firing rate of ventral nonoscillating neurons aligned to the start and end (dashed line) of fictive swimming. Firing rate increases when larvae start swimming. (e) Spike raster plot of a single V3 neuron for 80 swim cycles. Spikes are uniformly distributed throughout the VNR phase. (f) VNR-triggered average spike rate of V3 neurons showing no phase-dependent modulation in spike rate. Shaded area denotes SEM (n=87 cells). Data adapted from Böhm et al.^41^

Using the genetically encoded voltage sensor zArchon^40^ with a cylindrical lens-based light-sheet microscope, Böhm et al.^41^ investigated the activity patterns of glutamatergic neurons in the zebrafish spinal cord during fictive swimming. The direct measurement of membrane potential allowed the separation of two broad classes: in some neurons, membrane potential oscillated at a constant phase relative to the fictive swimming; in others, membrane potential showed clear spiking but no oscillations [Fig. 3(a)].

Measuring the relative phase of oscillating neurons, the authors demonstrated the left-right alternation and rostro-caudal delay of activity that propagates along the spinal cord during swimming [Figs. 3(b) and 3(c)]. These timing relationships had previously been accessible only by pooling data from single-cell recordings over many experiments.

A distinctive nonoscillating population of neurons in the very ventral spinal cord showed clear activity during fictive swimming [Fig. 3(d)], but their spikes were not phase-locked with the oscillations [Figs. 3(e) and 3(f)]. The ventral location and glutamatergic phenotype suggested them to be V3 (VeMe) neurons,^51^^,^^52^ which had so far been considered rhythmically active based on *ex vivo* recordings in rodents.^53^

Because V3 neurons are not oscillating, their activity likely does not directly contribute to the rhythm generation in the spinal cord, but their activity during swim episodes makes them an interesting candidate for fine-tuning motor output. By comparing the spiking activity of V3 neurons with the fictive motor output, the authors showed that the strength of the motor output correlated with the activity of V3 neurons, leading to the idea that V3 neurons act as a modulator of swimming strength in larval zebrafish.

This study nicely demonstrates the power of having access to subthreshold and spiking activity, as well as the necessity to measure activity at high temporal resolution to distinguish different activity types across a population of neurons.

### 4.3 Learning Computations in the Serotonergic System

**Fig. 4:**
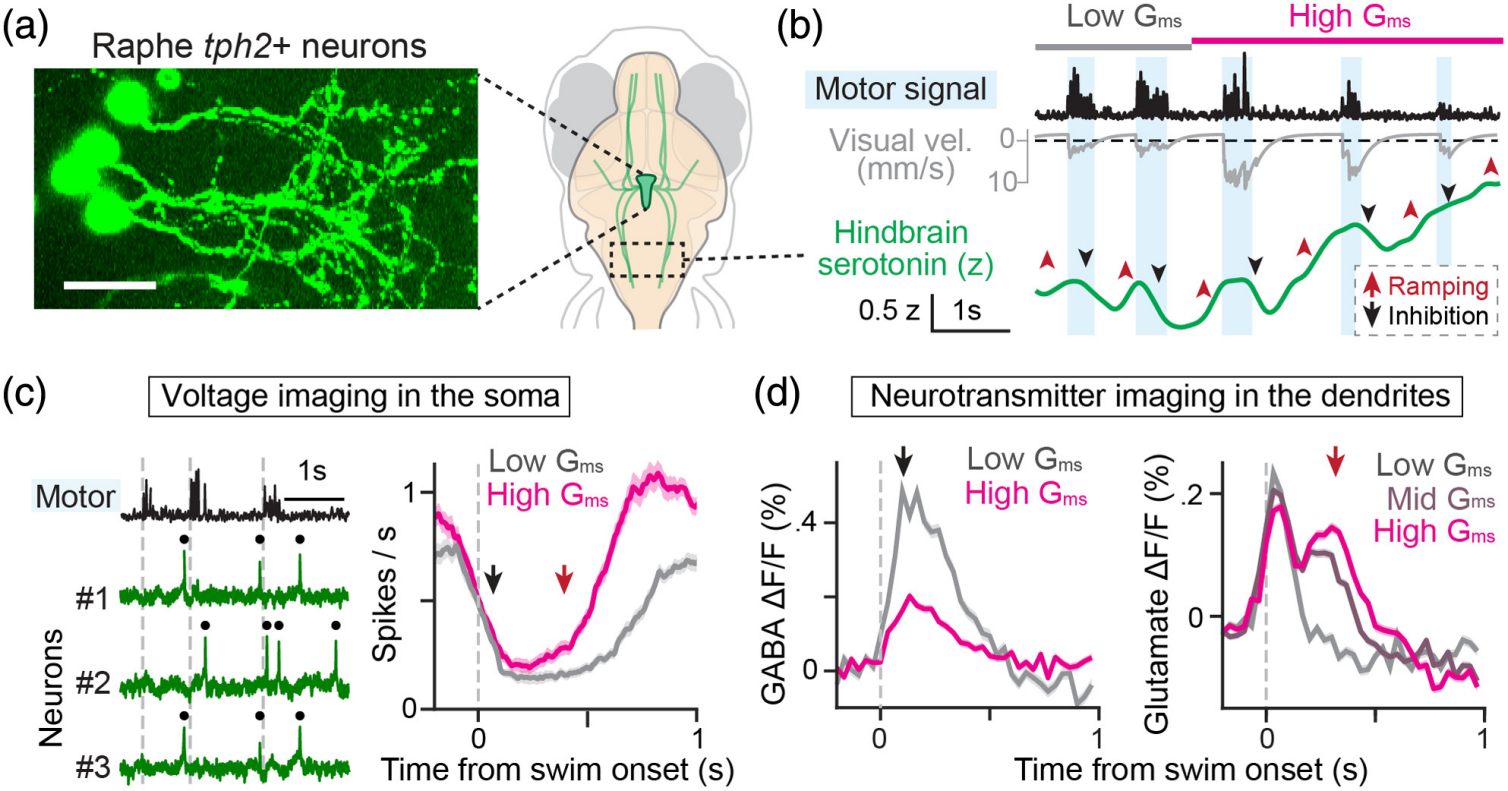
Internal computations of serotonergic neurons. (a) Left, morphology of raphe *tph2*+ serotonergic neurons expressing GFP. Scale bar, 20  μm. These neurons are larger than other neural types in the larval zebrafish brain (Figs. 1 and 2). Right, anatomical location of the raphe nucleus. (b) Dynamic modulation of axonal serotonin signals during motor vigor learning. In this task, the fish adapts its swim vigor according to the change in action effectiveness ($G_{\mathrm{ms}}$) of the virtual reality environment. Higher $G_{\mathrm{ms}}$ results in faster visual feedback during the optomotor response (gray) and attenuated swim signals (black). The serotonin signal exhibits initial inhibition during swimming (black arrow), followed by an increase immediately after (red arrow). At high $G_{\mathrm{ms}}$, the increases are stronger and more sustained, leading to ramping activity. (c) Left, swim signals and voltage signals from three simultaneously recorded neurons during motor vigor learning. Black dots represent recognized spikes, and gray lines denote the onset of swimming. Right, the action effectiveness ($G_{\mathrm{ms}}$) is encoded in the firing rate after post-inhibitory rebound. (d) Dendritic GABA (left) and glutamate inputs (right) under various $G_{\mathrm{ms}}$. The GABA peak (black arrow) coincides with swimming-induced suppression of serotonergic neurons, and the second glutamate peak (red arrow) precedes the post-inhibitory rebound of serotonergic neurons. Adapted from Kawashima, Wei, et al.^46^

A recent study examined the mechanisms of cellular-level computations of serotonergic neurons in the raphe nucleus during motor vigor learning.^46^ Serotonergic neurons play roles in various brain functions, including learning,^54^^,^^55^ mood control,^56^^,^^57^ and wakefulness.^58^^,^^59^ However, how they integrate multiple streams of behavioral information to shape their neural outputs remained elusive. Such a study is challenging in mammalian brains due to their anatomical depths. The optical access of zebrafish allowed imaging of a population of *tph2*+ serotonergic neurons [Fig. 4(a)] and clarified how they integrate learning-related information for each swimming event.^11^

To clarify the underpinnings of such neural computations, voltage imaging was used in combination with neurotransmitter indicators for serotonin, glutamate, and GABA. First, serotonin imaging revealed that serotonergic axons accumulate activity levels [Fig. 4(b)] in a manner consistent with a learning model involving error computations. Second, voltage imaging in the soma revealed a strong suppression at the onset of swimming, after which the membrane potential rebounds to a higher level if sufficient visual feedback is provided during swimming [Fig. 4(c)]. Lastly, glutamate and GABA imaging revealed the underlying, precise temporal sequence of motor-triggered inhibition and visually driven excitation [Fig. 4(d)]. The ablation of raphe GABA neurons impaired both inhibition and rebound, demonstrating the pivotal role of inhibitory inputs for integrating the learning signals in serotonergic neurons.^46^

These results demonstrate that voltage imaging is not only a useful method for recording millisecond-scale neural dynamics but also for providing a ground truth for integrating insights into how various drivers of neuronal dynamics act collectively to enable complex neural computations in the brain.

## 5 Development of Light-sheet Microscopy for Volumetric Voltage Imaging

Following the lead from calcium imaging, combining the small and transparent nature of zebrafish larvae with voltage imaging opens the possibility of recording single-neuron activity from the entire vertebrate brain—an achievement completely out of reach even only a few years ago. Even more than for single-plane imaging, the parallel, camera-based readout of light-sheet imaging becomes essential for the high recording speeds necessary. However, two major technical limitations have been a bottleneck for recording GEVI activity across volumes of zebrafish nervous tissue: focusing and camera speed. Being able to image several hundred volumes per second to resolve single action potentials requires focusing through the sample at very high speed. In addition, sequentially acquiring low-noise images in a severely photon-limited regime reaches the limit of even the most advanced commercially available sCMOS cameras. Two recent papers have made significant advances toward solving these issues.^47^^,^^60^

Unlike slower volumetric calcium imaging, moving the heavy detection objective to focus through the sample is not feasible at several hundred cycles per second. Remote focusing with a small reflective element at a remote imaging plane circumvents this issue. By forming an isotropically and uniformly magnified image in a remote plane (1.33× to match the water refractive index), the system has the advantage of only moving an optical element the size of the field of view (which is typically a few hundred micrometers) and offers the benefit of near aberration-free refocusing over an extended range away from the natural focal plane of the objective.^61^^–^^63^ However, using a polarizing beam splitter to separate incoming light from the detection objective and refocused light from the remote focusing plane, this method loses half the emitted photons.

### 5.1 Volumetric Voltage Imaging in the Spinal Cord

**Fig. 5:**
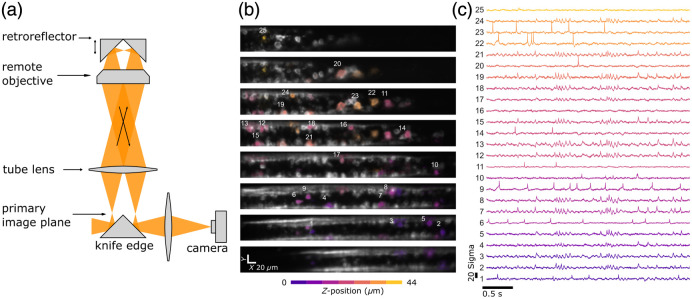
Volumetric voltage imaging with flipped image remote focusing. (a) Optic principle of the light-efficient remote focusing. Light from the microscope is reimaged on a retroreflector that can move rapidly and shift the focal plane before sending the light to the camera for image acquisition. (b) Sample optical planes through a 390×50×50  μm spinal cord volume. (c) Example traces of the cells marked in (b). The entire volume is imaged at 500  volumes/s and contains >100 active neurons. Adapted from Böhm and Judkewitz.^60^

Recently, a new version of remote focusing was introduced that doubles the light efficiency compared to the standard implementation.^60^ Using a microscopic retroreflector instead of a mirror and only refocusing half of the FOV, the image gets folded and flipped to the other side of the FOV and can be separated and relayed to the camera with a simple knife-edge mirror [Fig. 5(a)]. Using only reflective elements and discarding the polarizing beamsplitter in the imaging path doubles the light efficiency, albeit at the expense of only using half the FOV. Using a linear voice-coil motor, this method was able to scan a z-range of up to 150  μm at 500 Hz and was used to record an entire volume of larval zebrafish spinal cord (x=390  μm, y=46  μm, and z=50  μm), measuring activity of >100 neurons in parallel [Figs. 5(b) and 5(c)].

### 5.2 Voltage Imaging of Neurons distributed across the Entire Larval Zebrafish Brain at Cellular Resolution

Wang et al.^47^ developed and applied a remote-scanning light-sheet microscope (rsLSM) capable of recording the voltage of more than 25,000 neurons distributed throughout the entire zebrafish brain at a speed of >200  Hz.^47^ This work overcame several major challenges to image voltage signals at cellular resolution across a dense, whole-brain volume.

**Fig. 6:**
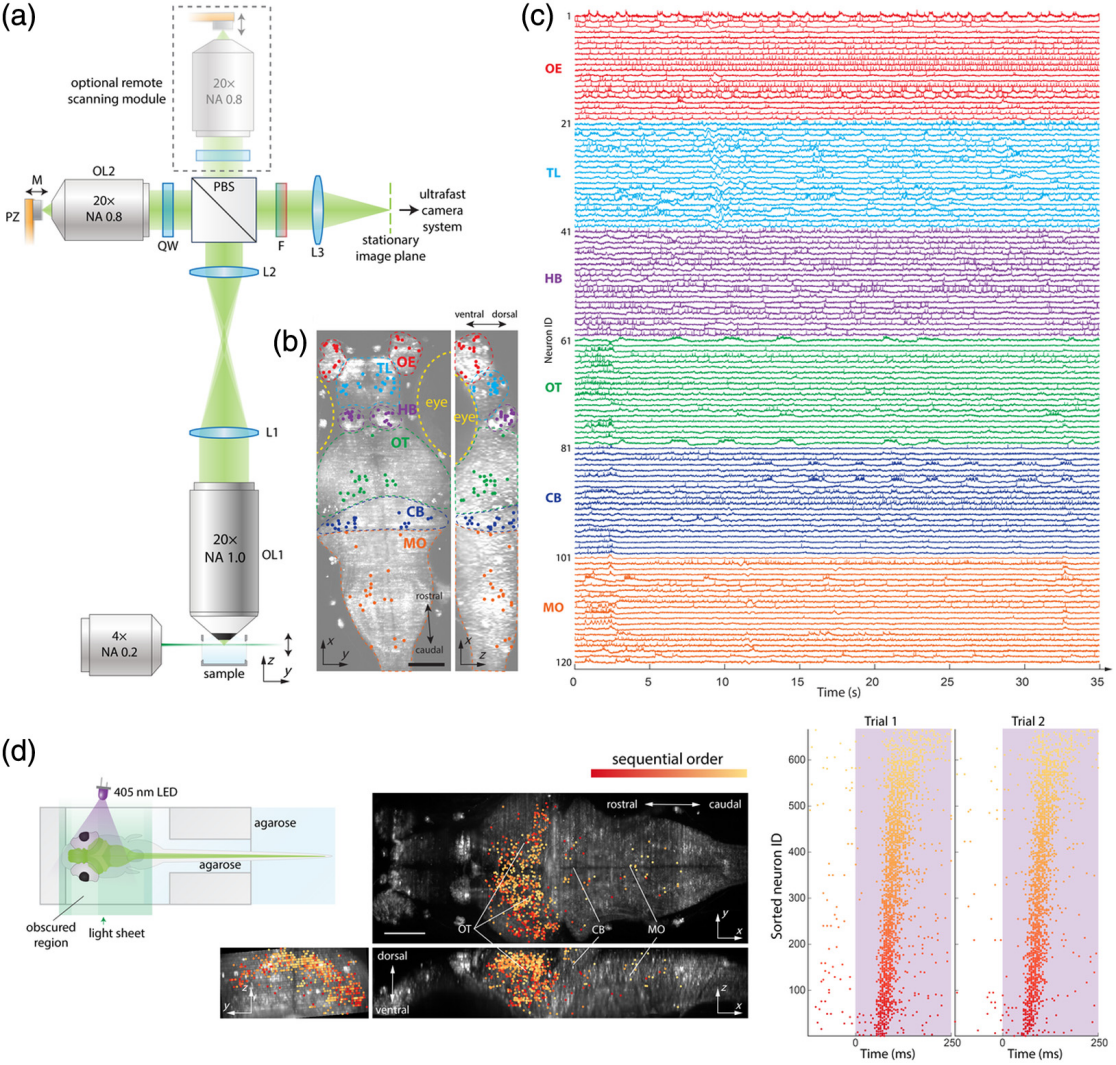
Volumetric voltage imaging of neurons distributed across the entire larval zebrafish brain. (a) Overview and operational principles of the remote scanning light-sheet microscope (rsLSM) optimized for imaging neuronal voltage across the whole larval zebrafish brain. A sample (illustrated as a point fluorescent bead) in a water chamber is illuminated from one side by a rapidly scanning light sheet delivered through an excitation objective. Fluorescence is collected orthogonally by a 20×, NA 1.0 water-immersion objective (OL1), then relayed through a 4f system (tube lenses L1 and L2) into a polarized beam splitter (PBS). The PBS directs one polarization into a quarter-wave plate (QW) and a 20×, NA 0.8 air remote objective (OL2), which forms real images. These images reflect off a mirror (M) translated by a piezo actuator (PZ) and are re-imaged by OL2. After passing the QW again, the polarization rotates by 90°, allowing fluorescence to pass through the PBS and an emission filter (F), then be focused by tube lens L3 into real images. The piezo-driven mirror moves synchronously with the scanning light sheet, keeping images of different z-planes sharply focused at the stationary focal plane of L3 (marked by the green dashed line), where an ultrafast camera records them. An optional second remote scanning module (gray dashed box) can be added to improve light efficiency. (b) Locations of the putative neurons (colored dots) for which activity traces are shown in (c), superimposed on the dorsal (left) and lateral (right) maximum intensity projections of the imaged brain. The putative neurons’ locations and brain regions are marked with the same colors as those of their corresponding activity traces in (c). Scale bar: 100  μm. (c) Spontaneous activity traces of 120 exemplar neurons from six brain regions, including olfactory epithelium (OE), telencephalon (TL), habenula (HB), optic tectum (OT), cerebellum (CB), and medulla oblongata (MO). (d) In the visual stimulation experiment, the light stimulus was delivered to one lateral side of the fish for 10 s in each trial. A population of neurons responding to stimulus onset exhibited a millisecond-precise, sequential activation pattern that reliably repeated across trials. The spatial locations of these neurons and the corresponding raster plot of their activity are shown. Scale bar: 100  μm. Adapted from Wang et al.^47^

First, to scan across the entire zebrafish brain (∼400×800×200  μm) at hundreds of volumes per second, the authors employed standard remote refocusing and a customized piezo-bender-driven lightweight mirror (2×2×1  mm) to axially scan a remote real image of the brain at hundreds of Hz [Fig. 6(a)]. The piezo was driven in open-loop mode to maximize scanning speed, which introduced piezo hysteresis. The authors corrected for this hysteresis by iteratively measuring and calibrating the piezo movement using a fast line imager. To capture single-plane images at high speeds, the authors built an ultrafast image-capture module that employed two CMOS cameras operating in parallel, each recording half of the field of view. This design doubled the effective frame rate, enabling single-plane acquisition at 6025 frames per second and yielding a volumetric imaging rate of 200.8 Hz while scanning 30 planes.

To maintain cellular resolution and high light efficiency to detect fast and small voltage fluctuations, the authors conducted optical simulations and optimization, which produced a pixel-size-limited resolution of 1.4  μm, approximately one-fifth of the typical zebrafish neuron soma diameter, across a cylindrical volume of $\Phi 900\times 200\;\mu m^{3}$. To maximize light collection, rsLSM used a 1.0 NA objective. Moreover, as an alternative to the retroreflector-based light-efficient remote focusing method as described above,^60^ the authors of rsLSM showed that emission light that would otherwise be lost in a standard remote focusing layout can be restored by adding a second remote-refocusing module. This strategy doubles light efficiency without compromising the field of view, while it increases the hardware complexity and requires extra nontrivial efforts in optical alignment, calibration, and synchronization.

Light-sheet imaging commonly suffers from “stripe” artifacts, which arise when partially opaque or refractive structures in the sample absorb, scatter, or refract the excitation light sheet.^64^^,^^65^ These structures cast striped shadows extending along the illumination direction. The rsLSM data exhibited such artifacts. The researchers demonstrated that these can be computationally removed by exploiting their characteristic spatiotemporal signatures. In addition, rapidly pivoting the light sheet within the illumination plane during each exposure averages out stripe patterns and further suppresses the artifact, as shown in previous studies.^65^^–^^68^

Together, these innovations enable rsLSM to map millisecond-scale voltage dynamics across the entire zebrafish brain [Figs. 6(b) and 6(c)]. In visual-stimulation experiments, rsLSM revealed that neurons activated at distinct times within millisecond-timescale sequences were spatially segregated [Fig. 6(d)]. For stimulation-evoked sequences mapped onto the optic tectum, neurons located more laterally tended to fire earlier. In addition, stimulus-independent burst sequences were mapped to the cerebellum and medulla. These sequences repeated across burst events and were consistent across individual fish. The results indicate that fast, distributed neural sequences may be widespread throughout the vertebrate brain and play important roles in neural computation, highlighting the value of high-speed volumetric voltage imaging.

## 6 Outlook

Light-sheet voltage imaging in larval zebrafish can leverage the unique advantages of the model system—a small and transparent nervous system—to measure unprecedented details about neuronal spiking and subthreshold modulation. As shown above, the technology is mature enough to already yield new insight into the function of defined neural populations, and thanks to new technological advancements, recording spiking activity from an entire brain is within reach. Single-objective light-sheet microscopy, such as swept confocally-aligned planar excitation (SCAPE)^69^ and oblique plane microscopy^70^^,^^71^ (OPM) holds further potential for samples where optical access is limited to one objective, although their use for voltage imaging has not been demonstrated so far. This would be especially beneficial for whole-brain imaging in older animals or other fish species such as *Danionella cerebrum*.^72^ Other new high-speed voltage imaging methods potentially useful in fish, such as light-field or fast confocal scanning, are discussed elsewhere in this issue.^73^

Technological improvements in voltage indicators and data processing pipelines will also help expand the use of voltage imaging. New rhodopsin indicators, such as Voltron2^74^ and monArch,^75^ will reduce the required intensity of excitation light and the microscope’s spatial duty cycle, enabling voltage imaging at a larger scale. New two-photon compatible indicators, such as JEDI-2P,^76^ ASAP5,^77^ and Jarvis,^78^ will expand voltage imaging into deeper, opaque tissues that light-sheet microscopy cannot reach. Data processing for voltage imaging is also fundamentally different from that for calcium imaging and has been a bottleneck. New signal extraction pipelines, including VolPy,^79^ SpikeyGi^80^ and ALI,^81^ offer versatile platforms for broader communities.

However, the relative complexity of setting up custom light-sheet microscopes and the idiosyncrasies of GEVIs still represent a significant hurdle for more widespread adoption. We summarize a few best practices and pitfalls in the hope of facilitating the adoption by more researchers. Due to the high recording speed, the number of photons per camera frame is severely limited. To achieve the best possible SNR, the microscope should therefore ideally operate at the shot noise limit, i.e., the major source of noise is from the stochasticity of photon emission. However, there are many additional and avoidable noise sources. Most commonly, these are either vibrations of the setup or intensity fluctuations of the excitation light. Intensity fluctuations can be due to electrical noise controlling or powering any of the equipment or to the characteristics of the excitation laser itself. Table 1 lists the ones we most commonly encounter.

## Data Availability

All data presented in this review are derived from previously published studies or preprints cited in the main text and figure captions, and are used with the authors’ approvals. Data availability follows the conditions stated in those original publications and preprints.
